# The ecological importance of moss ground cover in dry shrubland restoration within an irrigated agricultural landscape matrix

**DOI:** 10.1002/ece3.8843

**Published:** 2022-04-23

**Authors:** Rebecca Dollery, Mike H. Bowie, Nicholas M. Dickinson

**Affiliations:** ^1^ Department of Pest‐Management and Conservation Lincoln University Lincoln New Zealand

**Keywords:** biodiversity conservation, ecological restoration, ecosystem function, *Hypnum cupressiforme*, kānuka, *Kunzea*, mineral nitrogen, soil moisture

## Abstract

1. Kānuka (*Kunzea serotina*, Myrtaceae) dryland shrubland communities of the lowland plains of South Island (Te Wai Pounamu), New Zealand (Aoteoroa), contain a ground cover largely consisting of mosses, predominantly *Hypnum cupressiforme*. There has been no previous study of the role of mosses in this threatened habitat which is currently being restored within a contemporary irrigated and intensively farmed landscape that may be incompatible with this component of the ecosystem.
2. The aim of the present study was to investigate the influence of moss ground cover on hydrology, nitrogen (N) availability and vascular plant interactions, and in relation to nutrient spillover from adjacent farmland. Experimental work was a combination of glasshouse experiments and field‐based studies.
3. Extremes of soil temperature and moisture were found to be mediated by the moss carpet, which also influenced N speciation; available N declined with moss depth. The moss layer decreased the amount of germination and establishment of vascular plants but, in some cases, enhanced their growth. Spillover of mineral nitrogen and phosphate from farmland enhanced invasion of exotic grasses which may have benefited from conditions provided by the moss carpet.
4. *Synthesis*: We found the moss layer to be crucial to ecosystem functioning in these dry habitats with low nutrient substrate. However, when the moss layer is accompanied by nutrient spillover, it has the potential to increase exotic weed encroachment. Our results not only emphasize the importance of non‐vascular plant inclusion in restoration schemes but also highlights the importance of mitigating for nutrient spillover.

## Graphical abstract

This research investigated the importance of non‐vascular flora on ecosystem functioning in a dryland kānuka shrubland. We found the presence of a moss layer to be crucial in these dry habitats with low nutrient input with regard to maintenance of soil temperature, moisture, and available nitrogen inciting questions of inclusion in ecological restoration scheme planting.

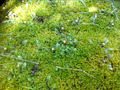

## 1 INTRODUCTION

Most mosses are poikilohydric and ectohydric, with limited ability to regulate water loss, acquiring moisture and nutrients via external conduction from surface water or dry deposition (rainfall, mist droplets, and airborne dust) (Proctor et al., 2007). In arctic, boreal, and arid ecosystems, prolific ground cover has enabled mosses to exert influence on soil temperature and nitrogen availability, and evapotranspiration and vascular plant interactions (Belnap, 2006; Betts et al., 1999; Bonan & Shugart, 1989; During & Tooren, 1990; Gornall et al., 2007). It has been suggested that mosses can be a potential source of nutrients and moisture to vascular plants, supplying leaked nutrients from their cells upon rehydration (Wilson & Coxson, 1999), or possibly through associations with mycorrhiza networks (Davey & Currah, 2006). The moss layer can also assist germinating seeds and seedlings, buffering harsh abiotic conditions and providing camouflage against seed predation (During & Tooren, 1990; Rayburn et al., 2012). Conversely, mosses may inhibit the germination of vascular plants by providing a barrier to the soil or rooting medium, drying the seed or maintaining excess moisture, sustaining temperatures too cold for germination, restricting light levels, and increasing the aboveground time available for seed destruction (During & Tooren, 1990; Head et al., 2004; Sohlberg & Bliss, 1987; Zamfir, 2000). Mosses can affect seedlings also by limiting available resources to surrounding vascular plants by sequestering nutrient precipitation inputs and accumulating organic matter (Cornelissen et al., 2007). An association with cyanobacteria fixes nitrogen which is retained by the moss rather than releasing it into the soil (Rousk et al., 2016). In the arctic, alteration of soil temperature and moisture have been shown to influence microbe activity and rates of mineralization and nitrification (Gornall et al., 2007).

**FIGURE 1:**
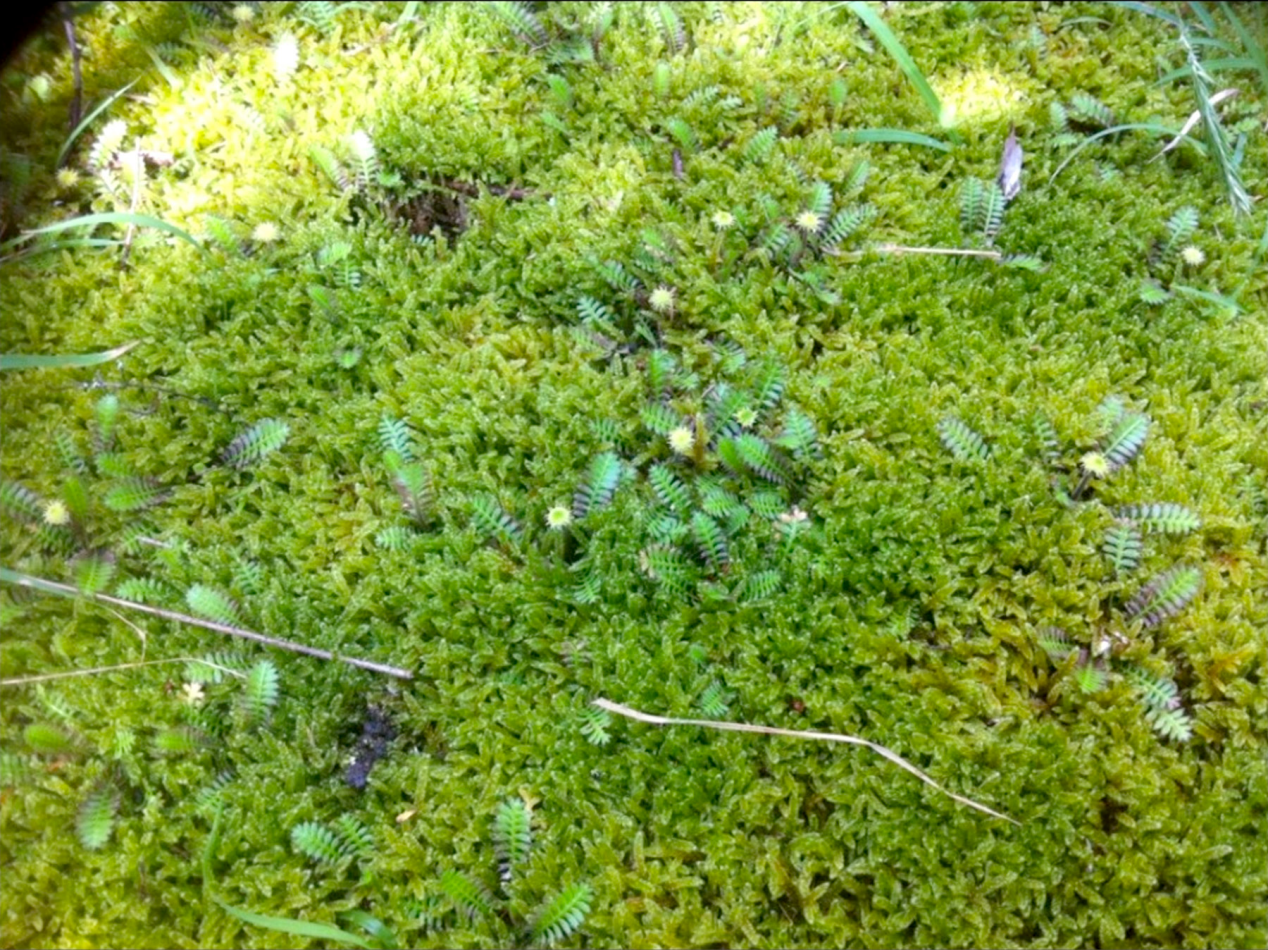
*Hypnum cupressiforme*forming a continuous carpet in kānuka‐dominated vegetation in the Eyrewell Forest, Canterbury. Growing within the moss is *Leptinella pusilla*, which is a creeping tufted perennial native forb of coastal to subalpine New Zealand (credit Bowie et al., 2016)

The role of mosses in ecosystem function is complex and often poorly understood (Bond‐Lamberty et al., 2011; Chamizo et al., 2016), and research surrounding the topic in New Zealand is almost entirely lacking (DeLucia et al., 2003; Michel et al., 2013) in spite of mosses accounting for >90% ground cover in many habitats (Pfeiffer, 2003). Even in some of the driest habitats, mosses, particularly *Hypnum cupressiforme*, form an almost continuous carpet; for example, under stands of the canopy shrub kānuka (*Kunzea serotina*, Myrtaceae, Figure 1) of lowland New Zealand (Beever, 1986; Macmillan, 1976; Molloy & Ives, 1972).

This research aimed to explore the role of the moss carpet within the kānuka stands associated with ecological restoration of a dryland habitat that has virtually disappeared from the contemporary irrigated intensively farmed landscapes of the lowland plains of eastern South Island in New Zealand (Bowie et al., 2016). In view of their natural ground cover prominence, it is likely that mosses have a functional role in the restoration of these ecosystems. It is also possible that these highly modified landscapes are unsuitable for the establishment of mosses. This has perspectives both in terms of the possible juxtaposition of these two land uses, and also whether restoration of dryland habitat has a role in provision of ecosystem services.

## 2 SITE AND METHODS

### 2.1 Site description

Field experiments were undertaken in two semi‐natural shrubland remnants located on the Canterbury Plains: an 18 ha privately owned remnant (Spencer‐Bower Remnant, SBR) (−43°42′91.13″S, 172°43′53.98″E) and a Department of Conservation (2.3 ha Scientific Reserve at Eyrewell) (ESR −43°38′31.56″S, 172°19′46.43″). Both were fenced but surrounded by irrigated dairy pasture since the 1990s for ESR and 2014 for SBR. These remnants comprised of canopies of kānuka (*Kunzea serotina* de Lange and Toelken, Myrtaceae) with a sparse understory containing native and adventive species of vascular plants, including prickly mingimingi (*Leptecophylla juniperina* C.M. Weiller, Ericaceae) and a pioneer species *Pomaderris amoena* Colenso, Rhamnaceae. In excess of 70% of ground cover consisted of bryophytes, mainly *H*. *cupressiforme* Hedwe. var. *cupressiforme*. The climate of the region is dry with a prevalence of strong northwesterly föhn winds, warm summers, cool winters, and <650 mm rainfall, leading to low humidity and high evapotranspiration rates (Macara, 2016). The predominant soil type at the sites is classified as Lismore, a free draining, shallow, stony, silty loam of low fertility (pH 5.1, nitrate 0.21 mg L^−1^, and ammonium 1.64 mg L^−1^, Olsen P 8.34 µg g^−1^).

### 2.2 Soil moisture and temperature sampling

All soil sample for analysis were extracted using an auger to gain an undisturbed soil sample. They were immediately placed into sealed bags and transported to the laboratory in an insulated box kept cool by the use of ice packs.

Soil moisture was measured during winter and summer months. Six depths of moss (0–1; 1.1–2; 2.1–4; 4.1–6; 6.1–8; and >8 cm) were identified and sampled within the ESR remnant. Three soil cores (5 cm diameter and 7.5 cm depth) were extracted and bulked under each moss depth prior to analysis. Gravimetric soil moisture content was determined on freshly collected field‐moist soil sieved to 4 mm (Blakemore et al., 1987). Soil temperature was measured beneath three different depths of moss (0, 3, and 9 cm; *n* = 4) for 19 days during the winter (June 2016) and summer (December 2016 and January 2017). HOBO^®^ Pro v2 weatherproof data loggers connected with 1.8 m cables to two external temperature probes were placed 5 cm beneath the soil surface and left *in situ* for 3 days to calibrate prior to data collection. Four data loggers failed during winter and two during summer under the 9 cm moss covering.

### 2.3 Available soil nitrogen analysis

Available nitrogen in the soil was determined from fresh soil samples collected under four depths of moss (0, 3, 6, and 9 cm) during the winter and summer months within ESR (*n* = 8) and under three depths of moss (0, 3, and 8 cm) from SBR (*n* > 13). Gravimetric moisture content was determined and a further 4 g subsample of field moist soil was extracted with 2 M KCl (potassium chloride) to estimate nitrate (${\text{NH}}_{3}^{‐}‐N$) and ammonium (${\text{NH}}_{4}^{‐}‐N$) concentrations using standard methodologies (Blakemore et al., 1987). The extracts were analyzed using Flow Injection Analyser (FIA) (Foss FIAstar 5000 triple channel with SoFIA software V1.30).

### 2.4 Glasshouse study

The effect of the moss layer on establishment and growth of vascular plants was investigated in a glasshouse randomized block design experiment. Seeds of native broom (*Carmichaelia australis*, *n* = 5) *K*. *serotina* and *P*. *amoena* (*n* = 10) were cleaned, inspected for damage under a microscope, and sprinkled onto substrate together in 20 trays, each containing one of five treatments (*n* = 4): Eyrewell soil (to 2 cm depth) and four *H*. *cupressiforme* moss layers of 2.5, 5, 7.5, and 10 cm depth on Eyrewell soil. Based on earlier findings, *P*. *amoena* seeds were pre‐treated in boiled water 12 h immediately prior to use. Trays were located inside an automatically watered mist tent located in a glasshouse. Seeds were monitored weekly for germination and left to establish for 6 months, apart from *K*. *serotina* which was left for 4 months. On completion of the experiment, plants were carefully harvested, washed, and oven‐dried (72 h, 60°C) and soil moisture content and KCl extractable nitrate and ammonium were determined.

### 2.5 Field study

Within each remnant, six transects of 80 m were identified starting at the fenceline, adjacent the irrigated pasture, and toward the opposite edge comprising a road (south–north, center point, approximately 50 m). Measurements were taken at the fenceline and at 10 m intervals. At each sampling point, plant composition and percentage cover, for vascular and non‐vascular plants, were collected using a 1 m^2^ quadrat. Three soil cores (2.5 cm diameter and 7.5 cm depth) were bulked from each quadrat. Soil samples were analyzed for moisture and KCL extractable nitrogen. The remainder of the samples were air dried, ground, and sieved to 2 mm. Plant available P was analyzed following the Olsen P method. The resultant Murphy Riley extractant was read at 880 nm on a Shimadzu UV mini‐1240 spectrophotometer. Soil pH was analyzed using a suspension of 10 g air‐dried soil with 25 mL dionized water left to stabilize for 12 h and analyzed using S20 SevenEasy™ pH meter.

### 2.6 Statistical analyses

All statistical analysis and graphical representation were carried out using Minitab^®^ (V 17.2.1) and SigmaPlot (V 12.3). Pearson's correlation was used to define the relationship between moss depth and soil moisture and temperature. Data were analyzed using ANOVA with post hoc Tukey HSD tests and two‐way ANOVA, and Kruskal–Wallis tests for germination and establishment experiments.

## 3 RESULTS

### 3.1 Soil moisture and temperature

**FIGURE 2:**
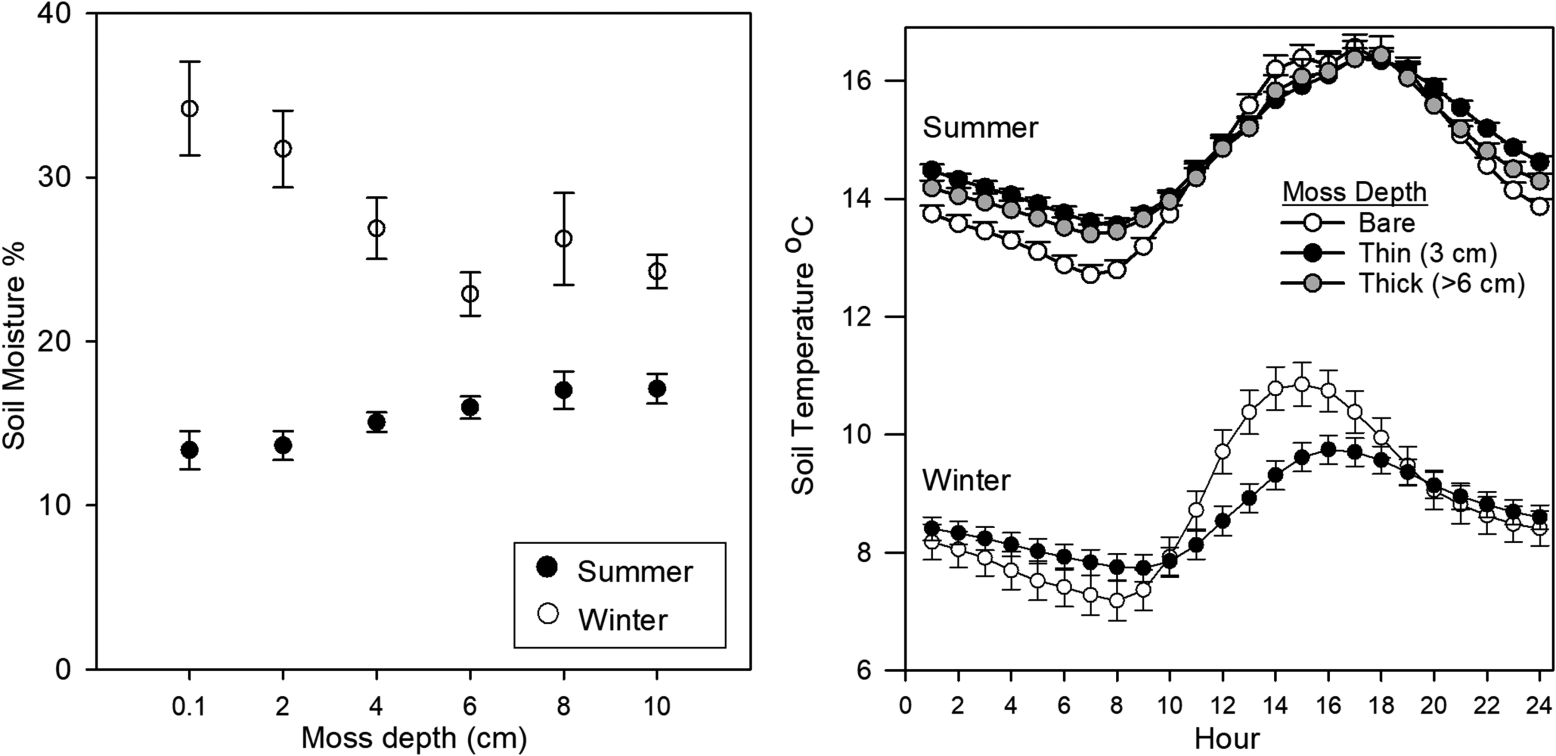
Mean gravimetric soil moisture and temperature (5 cm depth, ±SEM) beneath moss of increasing depth under the kānuka canopies in the field plots at SBR and ESR during the summer and winter months. Hours are based on a 24‐h clock. Means are significantly different (*p* < .05)

Gravimetric soil moisture was positively correlated with moss depth during the summer (*r* = .35, *p* < .001) and negatively correlated during the winter months (*r* = −.39, *p* < .001, Figure 2). Moisture was highest under thicker moss (*F*_5,79_ = 3.60, *p* < .01) in winter and vice versa in summer (*F*_5,107_ = 3.91; *p* < .005). There was marginally less fluctuation of soil temperature when the surface was covered by moss (Figure 2). In winter, temperatures were lower under moss cover in the daytime and higher during the night (*F*_1,908_ = 21.93; *p* < .001); mean diurnal fluctuation of bare soil was 1.3°C compared to 0.4°C under a thin layer of moss. During the summer, there was a diurnal fluctuation of 1.39°C for bare soil and 0.68°C for the thin moss cover (*F*_2,4554_ = 22.55; *p* < .001). In summer, no differences in daytime soil temperature could be attributed to the moss layers.

### 3.2 Soil nitrogen

**FIGURE 3:**
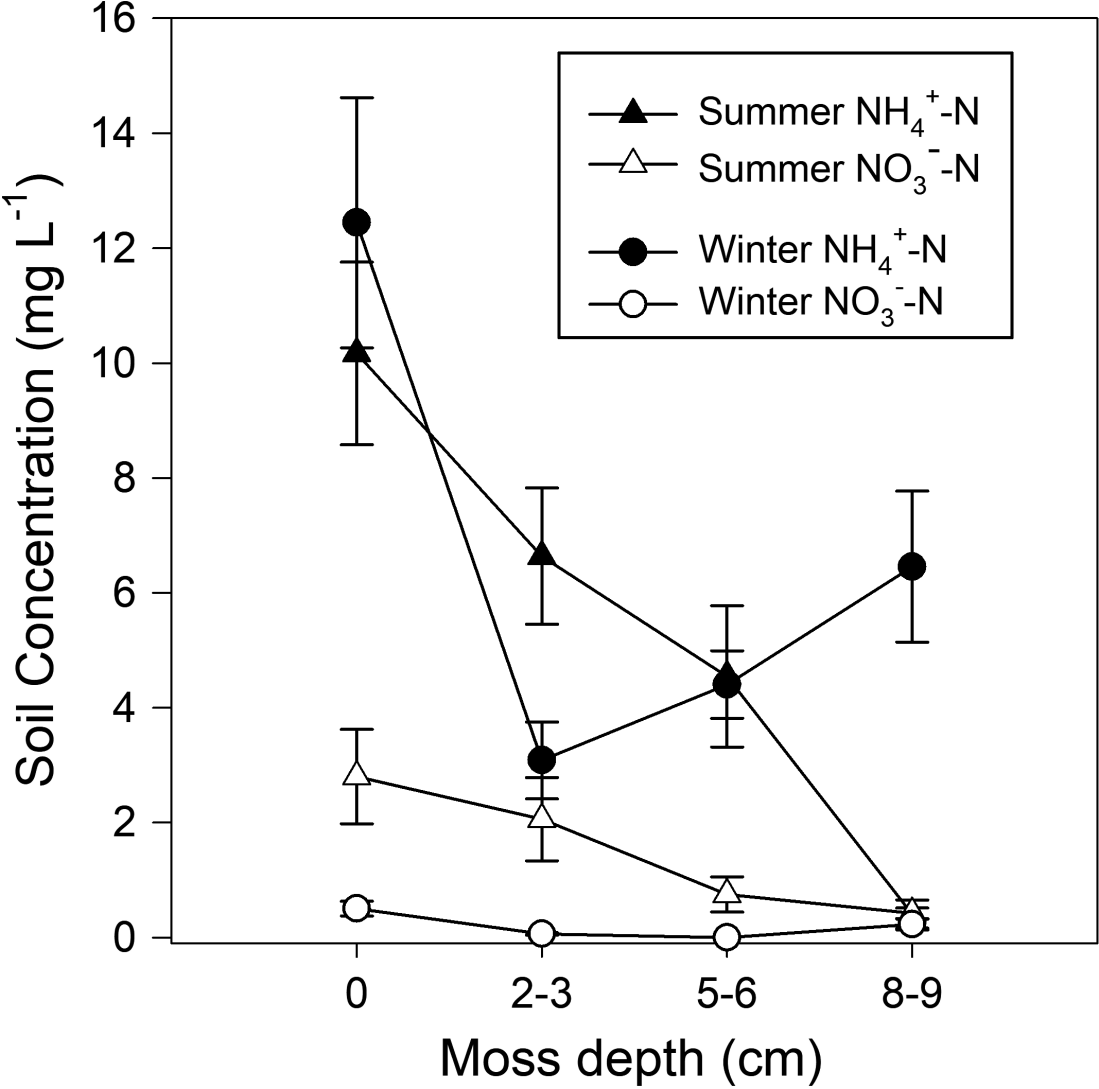
Mean nitrate and ammonium (±SEM) within soil samples under different depths of moss from under the kānuka canopies in the field plots at SBR and ESR during the summer and winter months. All means are significantly different (*p* < .05) except for summer ${\text{NO}}_{3}^{‐}‐N$

Soil ammonium and nitrate concentrations in bare soil were within the range expected for typical soils (2–30 mg L^−1^ and 1–20 mg L^−1^, respectively (Allen et al., 1989)), although nitrate levels were low and below 1.0 mg L^−1^ in winter (Figure 3). Both nitrate and ammonium were higher in bare soil than under a moss covering (*p* < .05).

### 3.3 Glasshouse study

**FIGURE 4:**
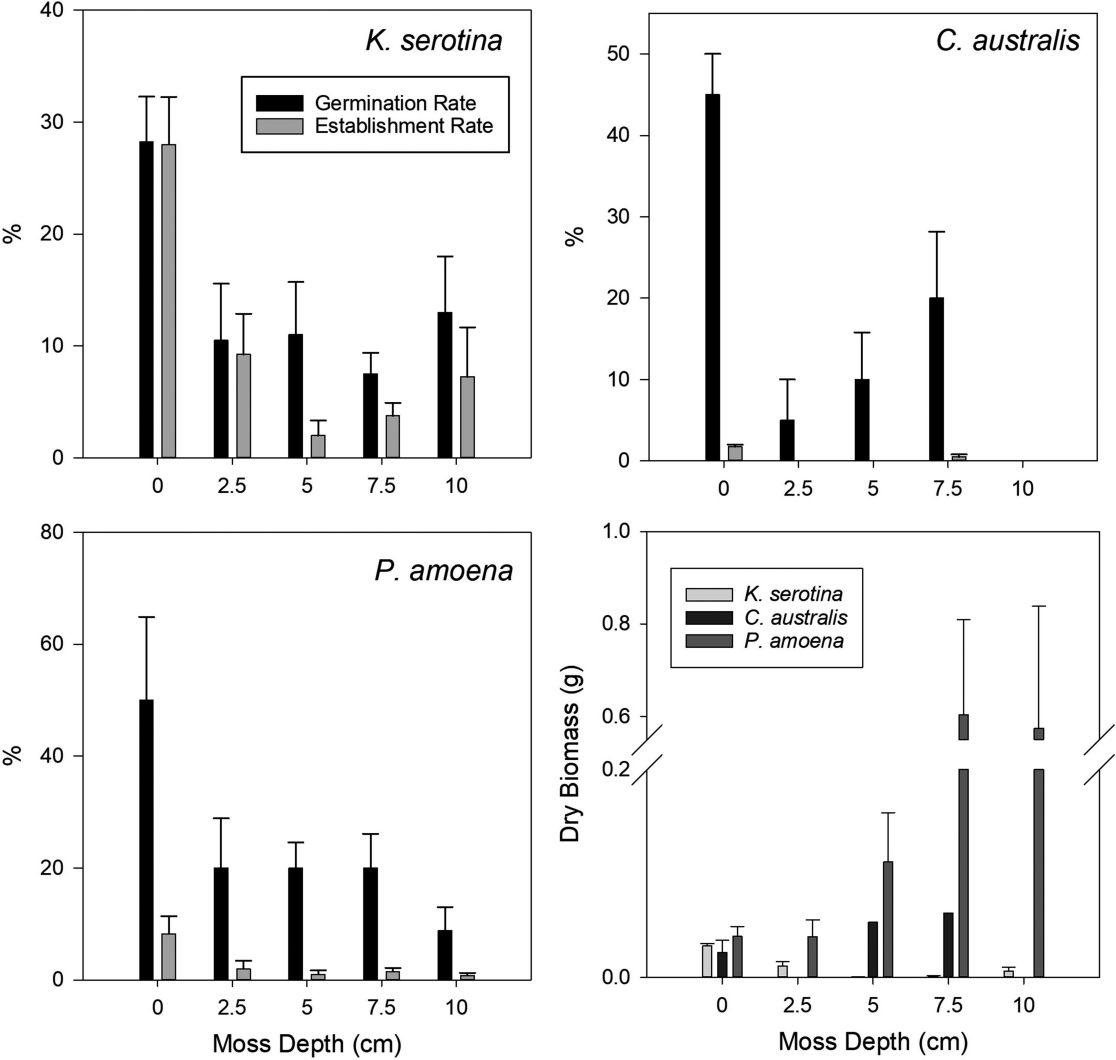
Germination and establishment rate (%) of seeds from three native species within moss of varying depths and total dry biomass (g) of all plants for each species in each treatment

By the end of the experimental period, germination rates were highest in bare soil for *C*. *australis* (H(4) = 13.06, *p* = .011) as were establishment rates for *C*. *australis* (H(4) = 15.54, *p* = .004) and *K*. *serotina* (H(4) = 10.75, *p* = .029) (Figure 4). *Kunzea serotina* biomass was highest in bare soil (H(4) = 36.09, *p* < .001), while moss cover increased biomass for *P*. *amoena*; particularly robust plants were observed beneath the deeper moss layers (H(4) = 19.89, *p* = .001). All seeds in the moss treatments germinated without contact with the soil and observation of rooting revealed that all plants were rooting into the dead moss layer beneath the live moss, as well as in the soil. Soil moisture and nitrate concentrations were not significantly different between the treatments but there was less nitrate under deeper moss (Table 1). *Carmichaelia australis* produced root nodules (for nitrogen fixation) in the moss treatments but not in bare soil.

**TABLE 1:** Soil moisture and mineral nitrogen concentrations under each moss depth on completion of the plant establishment experiment in the glasshouse

Treatment (moss depth cm)	% Soil Moisture (±SE)	${\text{NO}}_{3}^{‐}$Concentration mg L^−1^ (±SE)	${\text{NO}}_{4}^{‐}$Concentration mg L^−1^ (±SE)
0	15.57 (5.76)	0.41 (0.175)	3.11 (0.88)
2.5	38.40 (2.67)	0.62 (0.136)	4.90 (1.41)
5	25.68 (9.28)	0.46 (0.236)	3.26 (1.02)
7.5	23.46 (5.38)	0.19 (0.056)	2.191 (0.67)
10	26.86 (8.07)	0.17 (0.091)	2.73 (0.92)

### 3.4 Field study

**FIGURE 5:**
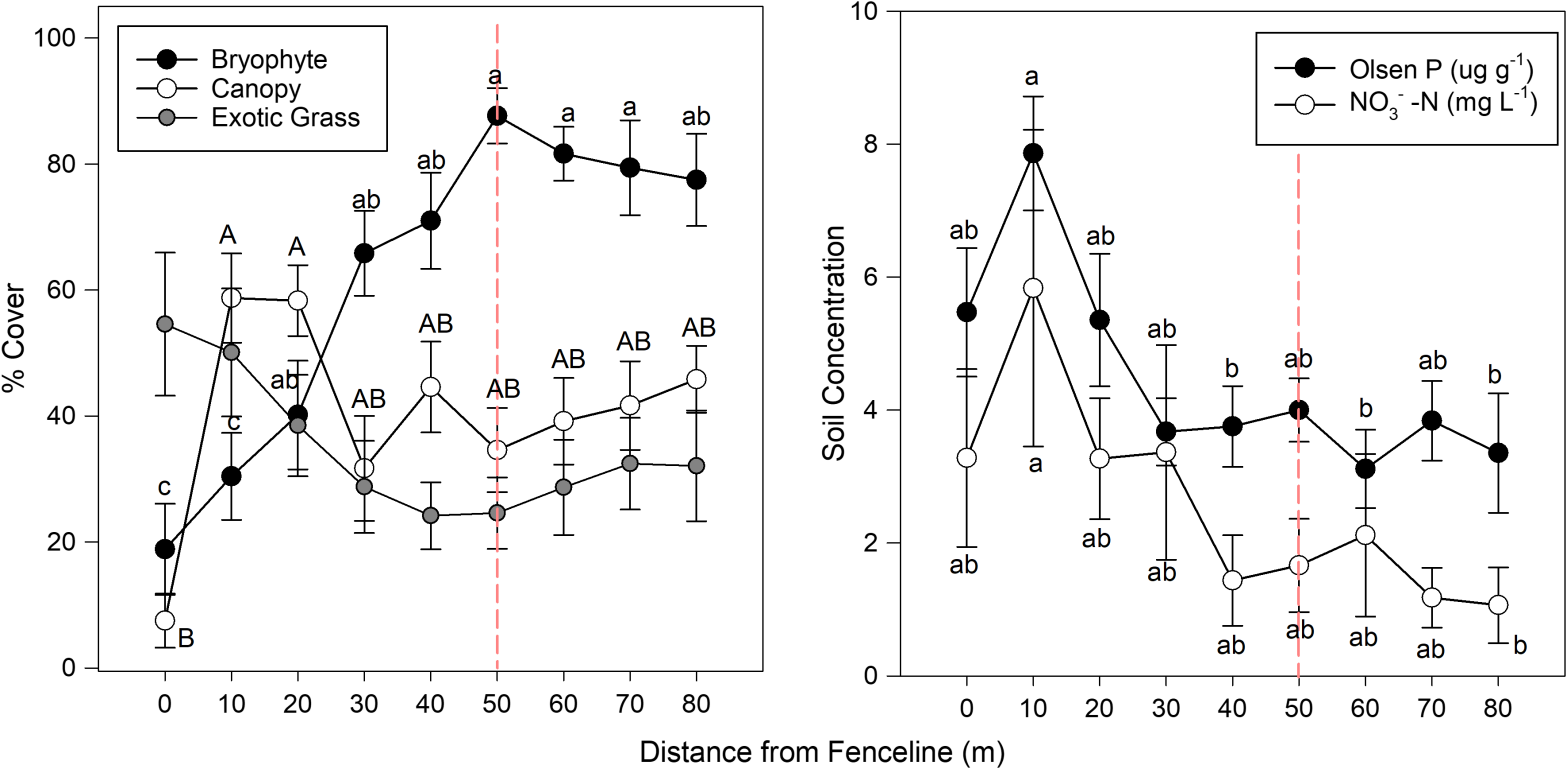
Relationship between biotic and abiotic parameters in relation to distance from fenceline adjacent irrigated pasture into the center of the dryland kānuka remnants (center point noted by red dotted line). Means that do not share a letter (either abc or ABC) are significantly different (*p* < .05), while no letters denote no significant difference

Clear patterns of plant cover were observed in relation to distance into both remnants, away from the fenceline and adjacent pasture (Figure 5). Moss cover increased with increasing distance from the pasture (*F*_(8,99)_ = 10.83, *p* < .001) while the canopy cover was lowest at the fenceline (*F*_(8,99)_ = 3.40, *p* = .002). There was a negative correlation between exotic grass cover and associated litter and distance into the center of the remnants, this was significant for ESR (H_(8)_ = 18.65, *p* = .017). Olsen P concentrations were highest at 0 and 10 m from the fenceline with a maximum mean of 7.9 µg g^−1^, and lowest near the middle of the remnants (*F*_(8,96)_ = 3.05, *p* = .004). Nitrate concentrations were highest closer to the fenceline and also lowest in the middle of the remnants (*F*_(8,82)_ = 2.16, *p* = .039). The soil pH and ammonium concentrations did not differ significantly throughout the remnants.

## 4 DISCUSSION

### 4.1 Soil moisture and temperature

This study showed that *H*. *cupressiforme* influences soil moisture and temperature within kānuka dryland shrubland soils by reducing annual and diurnal fluctuations. This is probably due to higher heat conduction capacity of water compared to the air which fills spaces within the moss (McLaren & Cameron, 1996) producing less heat transfer in dry summer months and increased transfer in wet winter months. The soil was wetter in summer under moss layers probably due to high water holding capacity of the moss moderating transfer of water to the soil, run‐off, and evaporation (Bu et al., 2015; Michel et al., 2013). Moss may also have harvested water vapor from the atmosphere. Deeper moss probably provides a physical buffer to ground frost, reducing heat transfer capacity in winter. In boreal forests, it has been reported that moss cover can intercept 23% of total rainfall, although this is thought to be much less in New Zealand forests (DeLucia et al., 2003; Price et al., 1997). Moss is metabolically active during the wetter winter months (Proctor et al., 2007) potentially further reducing soil moisture due to evapotranspiration.

Mediation of soil temperature and moisture may benefit vascular plants on these exposed sites on dry, well‐drained soils by improving water availability in the dry summer months. Vascular plants in the Lismore soil are known to reach wilting point at around 10–15% soil moisture (Drewitt, 1979); in this study, soil under a moss layer deeper than 6 cm was maintained above this range. These effects may become more significant with time since climate change is predicted to increase drought frequency and exacerbate soil moisture deficit in these habitats. However, competitive exclusion of adventive weeds in these dry habitats may be diminished by the retention of water availability (van der Wal et al., 2005).

### 4.2 Impact on nitrogen

The main process likely to be governing available soil nitrogen under moss ground cover in these habitats is interception and use by mosses. Soil nitrate and ammonium concentrations were lower under moss than in bare soil, and soil nitrate decreased with increasing moss cover. In cooler climates, this has been attributed to the effect of the moss cover on temperature and moisture, in turn affecting the microbes which facilitate ammonification and nitrification (Gornall et al., 2007). However, no extreme temperature variation was observed in this study.

*Hypnum cupressiforme*assimilates ammonium more readily than nitrate which explains a decrease in ${\text{NH}}_{4}^{‐}‐N$ during the winter months when the moss is metabolically active. In earlier studies, *H*. *cupressiforme* and other mosses have been shown to be effective at absorbing nutrients, including ${\text{NH}}_{4}^{‐}‐N$ in significant quantities, from wet and dry deposition, acting as a barrier to the soil and the rooting zone (Turetsky, 2003).

Soil ammonium was most reduced beneath moss at intermediate depths of 3 cm where it is possible that lack of the thick, dead plant layer underneath the moss allows for ${\text{NH}}_{4}^{‐}‐N$ uptake from the soil in addition to that deposited on the surface (Wang et al., 2014). An increase in moss depth increases the distance of actively growing shoots form the soil surface making soil ${\text{NH}}_{4}^{‐}‐N$ unattainable, potentially explaining the increase in ammonium concentrations beneath deeper moss layers (Bates, 1994). The present study also showed a reduction in nitrate soil concentrations under moss layers, probably due to nitrate formation being a product of microbial ammonification.

In addition to preventing ${\text{NH}}_{4}^{‐}‐N$, and therefore ${\text{NH}}_{3}^{‐}‐N$, reaching the soil by interception and utilization, mosses are also thought to recycle N within tissues, sequestering N for long periods of time and delaying release to soil and the vascular plant rooting zone (Turetsky, 2003). A study of feather mosses within a boreal forest found that mosses had been sequestering 1.8 kg N ha^−1^ year^−1^ for the past 5000 years (Lagerström et al., 2007). This is equivalent to approximately 1% of the annual fertilizer applications of N to dairy pasture in New Zealand (Chapman et al., 2018).

### 4.3 Interactions with vascular plants

Although exotic grasses and weeds are responsive to additions of nitrate and phosphorus (Blackshaw et al., 2004), an inhibitory effect was observed on moss cover. It is likely that moss reduction was a function of the competitive, shading, and smothering presence of the exotic grass and associated litter facilitated by increased nutrients (van der Wal et al., 2005).

Moss cover in the present study also negatively affected germination of the native species. Dormancy of seeds and inhibition of germination can be a response to far‐red light conditions altered by the moss cover thereby inhibiting germination (Van Tooren & Pons, 1988). Neither *K*. *serotina* nor *P*. *amoena* germinate as effectively in dark conditions (Burrows, 1996; Haines et al., 2007) and it is likely that the small seeds of each species dropped through the moss carpet following initial sowing, limiting the exposure to light. *Carmichaelia australis* can germinate in dark conditions (Grüner & Heenan, 2001) but germination rates were lower in the moss treatments; the moss may have acted as a barrier, preventing the radicle from reaching the soil (Jeschke & Kiehl, 2008). Although some mosses produce allelopathic substances that can inhibit vascular plant germination (P. Michel et al., 2011), it is not reported in *H*. *cupressiforme*. Those *C*. *australis* and *P*. *amoena* plants that established in the moss treatments resulted in higher biomass; reduced fluctuation in soil moisture and temperature under the moss layer may have influenced growth (Ren et al., 2010).

Mosses may use and sequester nitrogen restricting transfer to the vascular plant rooting zone, thereby constricting invasion. This was implied in the glasshouse study; nitrate can be inhibitive to nodulation for nitrogen fixation (Brewin, 1991) and *C*. *australis* only produced nodules in the moss layers, indicating less nitrate. Alteration of soil nutrition may also indirectly shape soil microbial communities (Delgado‐Baquerizo et al., 2017) which are central to biogeochemical cycles (Philippot et al., 2013). There are also suggestions that *H*. *cupressiforme* may have plant extracts which further modify microbial communities, potentially altering soil chemistry (Altuner et al., 2014).

Nutrient addition in the form of spillover into the remnants from the neighboring pasture was observed in the field study with regard to mineral nitrogen and Olsen P. The alteration of soil chemistry and potential influence on microbial communities was negatively correlated with moss cover and positively correlated with exotic grass cover species which are adapted to the more fertile soils (Meurk & Swaffield, 2000). The effects of the moss carpet particularly on soil moisture and temperature may have been beneficial to those individuals that established promoting smothering of mosses and a further alteration in soil chemistry (Hobbie, 2015). Therefore, although the moss layer may provide an effective tool in preventing spread of invasive plants by retaining a nutrient‐poor substrate, in the presence of increased nutrient deposition from nutrient spillover exotic weeds may initially benefit from abiotic conditions within the moss to further encroach and alter the habitat.

## 5 CONCLUSIONS

The effect of the terricolous moss cover in this low rainfall, typically nutrient‐poor environment, is clearly significant but it is complex and involves hydrology, nutrient cycling, and biotic interactions (Chamizo et al., 2016). Moss may be important in retaining soil moisture and maintaining low nutrient soil conditions which promote native species regeneration rather than exotic species encroachment. These functions support biodiversity, soil health, and nutrient sequestration, which are important supporting and regulating ecosystem services (Millennium Ecosystem Assessment, 2005). This importance should drive efforts to conserve the presence of moss in existing remnant habitats and indicate a requirement to incorporate it into ecological restoration schemes where ecosystem functioning is vital (Michel et al., 2013). However, where competitive exclusion has been mediated by nutrient spillover, the beneficial effects of the moss on the hydrological cycle may increase exotic species encroachment and alter soil chemistry further enhancing deterioration of the habitat. Therefore, ecological restoration in areas with soils of low nutrition should thoroughly consider and mitigate for the effects of nutrient spillover which could facilitate encroachment of weed species and decline in moss flora.

## CONFLICT OF INTEREST

The authors have no conflict of interests to declare.

## AUTHOR CONTRIBUTIONS

**Rebecca Dollery:** Conceptualization (equal); Data curation (lead); Formal analysis (equal); Funding acquisition (supporting); Investigation (lead); Methodology (lead); Project administration (lead); Visualization (equal); Writing – original draft (lead); Writing – review & editing (equal). **Mike H. Bowie:** Conceptualization (equal); Data curation (supporting); Funding acquisition (supporting); Investigation (supporting); Methodology (supporting); Project administration (supporting); Supervision (supporting); Visualization (supporting); Writing – review & editing (equal). **Nicholas M. Dickinson:** Conceptualization (equal); Formal analysis (supporting); Funding acquisition (lead); Investigation (supporting); Methodology (supporting); Project administration (supporting); Supervision (lead); Validation (lead); Visualization (equal); Writing – review & editing (equal).

## Data Availability

Data collected for this study are available in Dryad: https://doi.org/10.5061/dryad.83bk3j9t4.
